# Mechanism of *Astragalus membranaceus* Alleviating Acquired Hyperlipidemia Induced by High-Fat Diet through Regulating Lipid Metabolism

**DOI:** 10.3390/nu14050954

**Published:** 2022-02-23

**Authors:** Ling Wang, Wenya Zheng, Jinxin Yang, Anwar Ali, Hong Qin

**Affiliations:** 1 Department of Nutrition Science and Food Hygiene, Xiangya School of Public Health, Central South University, 110 Xiangya Road, Changsha 410078, China; wl202108@csu.edu.cn (L.W.); wenyazheng0326@163.com (W.Z.); yangjinxin20212021@163.com (J.Y.); 2 Department of Epidemiology and Health Statistics, Xiangya School of Public Health, Central South University, 110 Xiangya Road, Changsha 410078, China; 206908003@csu.edu.cn

**Keywords:** high-fat diet, *Astragalus membranaceus*, acquired hyperlipidemia, lipid metabolism, network pharmacology, AKT1, VEGFA, CCND1, ESR1

## Abstract

*Astragalus membranaceus* (AM) is a food and medicinal homologous plant. The current research is aimed to investigate the beneficial effects and mechanisms of AM in treating acquired hyperlipidemia. The network pharmacology and bioinformatics analysis results showed 481 AM-related targets and 474 acquired hyperlipidemia-associated targets, and 101 candidate targets were obtained through the intersection, mainly enriched in endocrine resistance, AGE-RAGE in diabetic complications and p53 signaling pathways. *Quercetin*, *kaempferol*, *calycosin*, *formononetin and isorhamnetin* were determined as the candidate active components of AM in the treatment of acquired hyperlipidemia. Moreover, key targets of AM, namely, AKT serine/threonine kinase 1 (AKT1), vascular endothelial growth factor A (VEGFA), cyclin D1 (CCND1) and estrogen receptor 1 (ESR1), were screened out, which were closely related to adipogenesis, fatty acid metabolism and bile acid metabolism. The subsequent animal experiments showed that AM extract treatment improved the lipid profiles of the high-fat diet (HFD)-fed mice by reducing lipogenesis and increasing lipolysis and lipid β-oxidation, which were associated with the downregulating of AKT1 and CCND1, and the upregulating of VEGFA and ESR1 in liver and adipose tissue. Overall, AM alleviated acquired hyperlipidemia through regulating lipid metabolism, and AKT1, VEGFA, CCND1 and ESR1 might be the key targets.

## 1. Introduction

With the change of diet and lifestyle, the incidence rate of acquired hyperlipidemia increases year by year. Acquired hyperlipidemia refers to abnormal lipoprotein metabolism in plasma [1], which is regarded as a significant risk factor for many metabolic diseases, such as non-alcoholic fatty liver disease, atherosclerosis and diabetes [2,3]. Due to the low toxic side effects, natural active substances derived from plants or food have been highlighted in treating chronic metabolic disease [4,5]. These plant extracts can regulate lipid metabolism through multi-component and multi-target regulatory networks [6,7]. Therefore, it is of high healthy value to explore the application of plants and their compounds in acquired hyperlipidemia [8,9]. *Astragalus membranaceus* (AM) sweet in flavor and warm in nature, is a commonly used plant with a homology of food and medicine belonging to Astragalus in Leguminosae [10]. AM contains flavonoids, saponins, polysaccharides and other components, which exert essential functions, such as immunomodulating, antioxidant, anti-inflammatory and anticancer [11,12].

Nevertheless, there are few studies on the role of AM in acquired hyperlipidemia. Of note, Astragaloside IV, a natural saponin extracted from AM, was unfolded to attenuate liver injury in type 2 diabetes by regulating the AMP-activated protein kinase (AMPK)/mammalian target of rapamycin (mTOR)/autophagy pathway, which could alleviate insulin resistance and dyslipidemia [13]. *Formononetin*, an AM component, alleviated atherosclerosis in ApoE-/- mice by regulating the interaction between krüppel-like factor 4 (KLF4) and steroid receptor RNA activator 1 (SRA) [14]. Moreover, it was reported that Astragalus polysaccharides could contribute to a decline in plasma cholesterol via a mechanism different from statins [15]. In view of the above findings, it is of a certain value to explore the main active components and targets of AM in the treatment of acquired hyperlipidemia. Meanwhile, further integration and analysis based on the existing evidence may help to explore further the specific mechanism of AM in the treatment of acquired hyperlipidemia.

Therefore, in this study, we screened the common targets of AM and acquired hyperlipidemia through network pharmacology and bioinformatics technology. An “AM-component-target” network was constructed to clarify the active components, main signal pathways and key targets of AM in the treatment of acquired hyperlipidemia. Further experiments were conducted to verify the effects of AM on lipid metabolism and dyslipidemia in high-fat diet (HFD)-fed mice and the effects on regulating the expression of key targets. The outcomes from this study can provide a new theoretical basis for AM in treating hyperlipidemia induced by HFD and crucial information for exploring natural functional components in AM.

## 2. Materials and Methods

### 2.1. Acquisition of Active Components and Targets of AM

Based on systematic pharmacological research methods, the traditional Chinese medicine systems pharmacology database and analysis platform (TCMSP) (https://tcmspw.com/tcmsp.php, last accessed on 20 February 2021) database integrates the active components, potential targets, related diseases and pharmacokinetic data of plant compounds. Oral availability (OB) and drug-like properties (DL) are important indicators for evaluating pharmacokinetic absorption, distribution, metabolism, and excretion (ADME). So, the active components of AM were searched through the TCMSP database according to the screening criteria of OB ≥ 30% and DL ≥ 0.18. Meanwhile, the corresponding targets were downloaded. Furthermore, the official gene symbols corresponding to the targets were retrieved (the “species” was limited to “Homo sapiens”) through the database UniProtKB (https://www.uniprot.org/, last accessed on 3 June 2021). In addition, the public Comparative Toxicogenomics Database (CTD) (http://ctdbase.org/, last accessed on 3 June 2021) and STITCH database (http://stitch.embl.de, last accessed on 3 June 2021) were used to retrieve the targets corresponding to the active components of AM. The retrieval results of TCMSP, CTD and STITCH databases were intersected to obtain the related targets of AM.

### 2.2. Microarray Analysis (Acquisition of Acquired Hyperlipidemia/Obesity-Related Targets)

Three mRNA expression microarray datasets of acquired hyperlipidemia were retrieved through the Gene Expression Omnibus (GEO) database. The GSE111412 microarray dataset contained adipose tissue samples from three normal mice and three mice with HFD; the dataset was equipped with platform annotation file GPL6887-10677. The GSE139601 microarray dataset had adipose tissue samples from 12 mice with a low-fat diet (LFD) and 11 mice with HFD; the dataset was equipped with a platform annotation file GPL16570-1802. The GSE15653 microarray dataset contained liver tissue samples from 5 normal individuals and 4 obese patients; the dataset was provided with platform annotation file GPl96-57554. Subsequently, the “limma” package in R language was applied for differential analysis; with *p* < 0.05 indicating the significance used as the screening condition, differentially expressed mRNAs were screened. The target genes related to acquired hyperlipidemia were searched via the CTD database, and the screening condition was set at inference score ≥ 50. The jvenn tool (http://jvenn.toulouse.inra.fr/app/example.html, last accessed on 4 June 2021) was used to further intersect the acquired results with the retrieval results from the CTD database after the analytical findings of the three datasets were intersected. The disease-related targets were thus obtained, and the Venn diagram was drawn.

### 2.3. Acquisition of Common Drug and Disease Targets

Using the JVENN tool, the above AM-related and disease-related targets were intersected to obtain the expected targets, namely candidate targets, for subsequent analysis.

### 2.4. Enrichment Analysis Using Gene Ontology (GO) and Kyoto Encyclopedia of Genes and Genomes (KEGG)

GO and KEGG enrichment analyses were performed on candidate targets using the “clusterprofiler” package in R language. A bubble diagram of enrichment analyses on cell components and molecular functions and a complete circle diagram of KEGG enrichment analysis on biological processes were drawn.

### 2.5. Visualization of Network Pharmacology and Construction of a Protein-Protein Interaction (PPI) Network

A network of AM-component-target was constructed using the Cytoscape software (v3.8.2). The degree value represents the number of connections between a node and other nodes in the regulatory network. Colors were used to represent the degree value, and the active components and targets were sorted according to the degree value. In addition, the interaction network of the candidate targets was obtained through the STRING database (https://string-db.org, last accessed on 7 June 2021), with the “species” condition limited to “Homo sapiens”. To create a regulatory network, the data were loaded into the Cytoscape software, and the targets were sorted according to the degree value to display the top 15. Based on the data from the GSE139601 dataset, correlation analysis of the mRNA expression of the 15 targets was performed using online bioinformatics tools (http://sangerbox.com/Tool, last accessed on 7 June 2021). Finally, to obtain the key targets, the jvenn tool intersects the top 15 targets according to the degree value in the above two networks. 

### 2.6. Gene Set Enrichment Analysis (GSEA) of Key Targets

Based on the GSE13960 microarray dataset, GSEA was performed utilizing the GSEA software (v4.0.0) according to the key targets’ high and low expression. Regarding the HALLMARK dataset, the pathways with significant differences were screened with |normalized enrichment score (NES)| > 1, nominal (NOM) *p*-value < 0.05 and false discovery rate (FDR) q-val < 0.25 as the threshold and the enrichment plot was delineated. 

### 2.7. Chemicals and Reagents

AM extract was produced by Sichuan Qi Li Pharmaceutical Company and purchased from the Chinese Medicinal Herbs Company (Beijing, China). Beijing Tongke Yuanda Pharmaceutical Technology Company evaluated the quality of AM according to the number of Huangqi (AM) batch number “Huangyi Decoction Piece Quality Standard” (STP-C/019-00). Triglyceride (TG, A110-1), total cholesterol (TC, A111-1), low-density lipoprotein cholesterol (LDL-C, A113-1), high-density lipoprotein cholesterol (HDL-C, A112-1), free fatty acid (FFA, A042-1) and glycerol assay kits (F005) were acquired from the Institute of Jiancheng Bioengineering (Nanjing, China). Protease inhibitor was provided by Dingguo Changsheng Biotechnology Co., Ltd. (Beijing, China). The bicinchoninic acid (BCA) protein assay kit was purchased from Beyotime Biotechnology, Inc. (Shanghai, China). Antibodies against fatty acid synthase (FASN, ab99359), adipose triglyceride lipase (ATGL, ab99532), carnitine palmityl transferase 1α (CPT1-α, ab234111), AKT serine/threonine kinase 1 (AKT1, ab81283), vascular endothelial growth factor A (VEGFA, ab1316), cyclin D1 (CCND1, ab16663), estrogen receptor 1 (ESR1, ab241557) and glyceraldehyde-3-phosphate dehydrogenase (GAPDH, ab9485) were purchased from Abcam (Cambridge, UK), secondary antibody horseradish peroxidase (HRP) goat anti-rabbit IgG (AS014) were obtained from Abclonal, Inc. (Boston, MA, USA).

### 2.8. Animals and Diet

Six- to eight-week-old male C57BL/6J mice were acquired from Central South University (Changsha, China). The mice were kept in controlled conditions of optimum temperature (22 ± 2°C) with 12 h/12 h light/dark cycle and had access to food, tap water and libitum. First, the mice were divided into two groups: (1) the mice (*n* = 10) as the normal diet group (ND) were fed with ND, and (2) the mice (*n* = 20) as the HFD group to establish the acquired hyperlipidemia model that was fed with HFD (60 kcal% fat; rodent diet D12492). Research Diets (New Brunswick, NJ, USA) prepared all diets. After 8 weeks, body weight and serum lipid were measured to identify the acquired hyperlipidemia model. Then, the bulkiest 14 mice were carefully chosen for the succeeding experiment and categorized into two weight-matched groups: the HFD group (HFD, *n* = 7) and the HFD + AM group (HFD + AM, *n* = 7). HFD or ND diet was given to all the mice for eight more weeks. AM was administered once daily at a dose of 0.1 mL/10 g body weight via oral gavage in a vehicle (0.5% carboxyl methylcellulose). Mice in the HFD group were gavaged with the same volume of the vehicle only.

### 2.9. Collection of Serum and Tissue

Serum samples were taken from the femoral artery after 16 weeks and prepared by centrifugation. Afterward, serum was stored at −80 °C for further analysis. Subsequently, mice were euthanized for tissue collection. Liver and white adipose tissue (WAT) were divided into two parts, either immediately fixed in 4% formalin solution or quickly frozen in liquid nitrogen.

### 2.10. Serum Biochemical Analyses

TG, TC, LDL-C, and HDL-C levels were determined using chemical reagent kits, as directed by the manufacturer.

### 2.11. Liver Tissue and WAT Biochemical Analyses

The liver tissue and WAT were homogenized with physiological saline, followed by centrifugation (2500 rpm for 10 min at 4 °C). The levels of TC and TG in liver tissue the content of FFA and glycerol in WAT were determined using chemistry reagent kits.

### 2.12. Western Blot Analysis

Liver tissue and WAT were homogenized in a cold lysis solution containing protease inhibitor. Next, these homogenates were centrifuged for supernatants collection, 12,000 rpm for 15 min at 4 °C. Then, the protein concentrations were examined using a BCA protein assay kit. SDS-PAGE separated these proteins under reducing conditions and then transmitted them to polyvinylidene fluoride membranes. Membranes were incubated for 1 h at room temperature in the blocking buffer with 0.1% Tween-20 and 5% non-fat milk, then immunoblotted overnight on a rocking platform at 4 °C with primary antibodies: FASN, ATGL, CPT1-α, AKT1, VEGFA, CCND1, ESR1 and GAPDH. These membranes were then washed and incubated in secondary antibodies at room temperature for 1 h. The chemiluminescence imager visualized the bands on membranes (Tanon-5500, Tanon Science & Technology Co., Ltd., Shanghai, China) with ECL. The target bands densities were analyzed by using Image J software.

### 2.13. Statistical Analysis

Statistical analysis was performed by using the SPSS 20.0 statistical program. All the data were expressed as the mean ± standard deviations (SD). Comparison among groups was performed using one-way variance analysis (ANOVA). Significant differences between the parameters used the least-significant difference t-test (LSD-t). Results were evaluated statistically significant at *p* < 0.05.

## 3. Results

### 3.1. Network Pharmacology and Bioinformatics Analysis Process

**Figure 1:**
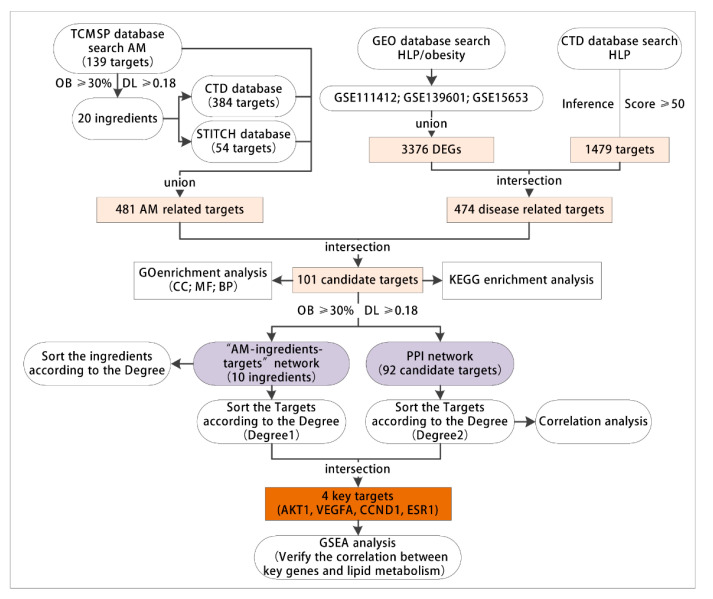
The flow chart for network pharmacology and bioinformatics analysis. TCMSP, the traditional Chinese medicine systems pharmacology database and analysis platform. AM, *Astragalus membranaceus*. GEO, the Gene Expression Omnibus; CTD, the public Comparative Toxicogenomics Database; HLP, hyperlipidemia; DEGs, differentially expressed genes; GO, Gene Ontology; CC, cell components; MF, molecular functions; BP, biological processes; OB, oral availability; DL, drug-like properties; KEGG, Kyoto Encyclopedia of Genes and Genomes; PPI, protein-protein interaction; AKT1, AKT serine/threonine kinase 1; VEGFA, vascular endothelial growth factor A; CCND1, cyclin D1; ESR1, estrogen receptor 1; GSEA, gene set enrichment analysis.

This project explores the key targets of AM to alleviate hyperlipidemia induced by HFD through network pharmacology and bioinformatics technology. The specific screening process is as follows. (1) The active components and targets of AM were retrieved through the TCMSP, CTD and STITCH databases. (2) The acquired hyperlipidemia/obesity-related microarrays were searched through the GEO database, and the differentially expressed genes (DEGs) were screened. The acquired hyperlipidemia-related targets were searched through the CTD database. The genes screened from the two databases were intersected to obtain the disease targets. (3) The drug and disease-related targets were intersected to obtain candidate targets. (4) On the putative targets, GO and KEGG enrichment analyses were performed to explore the possible ways of AM to alleviate lipid metabolism and dyslipidemia. (5) The network of AM component targets was developed; the targets were sorted according to the degree value (degree 1), and the key active components were identified. (6) The PPI network of candidate targets was constructed. The targets were sorted according to the degree value (degree 2), followed by the intersection of degree 1 and degree 2 to determine the key targets. (7) Based on the acquired hyperlipidemia related microarray data, gene set enrichment analysis (GSEA) was performed on the high and low expression of the key targets to verify the possibility of AM in alleviating acquired hyperlipidemia induced by HFD through the key targets (Figure 1).

### 3.2. AM Exerted Pharmaceutical Effects by Regulating 481 Related Targets through 20 Active Components

The active components and targets in AM were searched through the TCMSP database, and a total of 87 active components and 139 targets were thus obtained. According to the screening criteria of OB ≥ 30% and DL ≥ 0.18, 20 active components in AM were further obtained (Table 1). In addition, we searched the 20 active components one by one through the CTD and STITCH databases, and 384 and 54 targets were obtained, respectively. The retrieval results of TCMSP, CTD and STITCH databases were intersected, which received 481 AM-related targets.

### 3.3. 474 Targets Were Closely Related to the Occurrence and Development of Hyperlipidemia Induced by HFD

**Figure 2:**
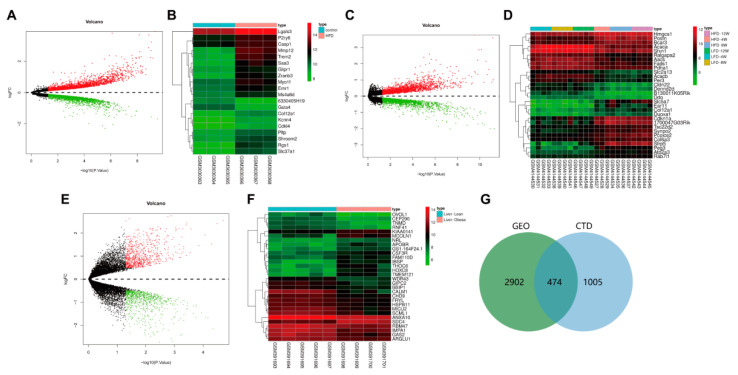
GEO and CTD databases-based screening of disease targets. (**A**) The volcano map of DEGs was analyzed through the GSE111412 dataset. (**B**) The heatmap of the top 30 DEGs in the GSE111412 dataset (control group: *n* = 3; HFD group: *n* = 3). (**C**) The volcano map of DEGs analyzed through the GSE139601 chip. (**D**) The heatmap of the top 30 DEGs in the GSE139601 dataset (LFD-4 w group: *n* = 4; LFD-8 w group: *n* = 4; LFD-12 w group, *n* = 4; HFD-4 w group: *n* = 3; HFD-8 w group: *n* = 4; HFD-12 w group, *n* = 4). (**E**) The volcano map of DEGs was analyzed through the GSE15653 dataset. (**F**) The heat map of the top 30 DEGs in the GSE15653 dataset (liver-lean group: *n* = 5; liver-obese group: *n* = 4). Red indicates upregulated genes in Panel (**A**,**C**,**D**) and green indicates downregulated genes. (**G**) The Venn diagram of intersection of analytical results from GEO and CTD databases.

Subsequently, we searched the acquired hyperlipidemia/obesity-related microarray dataset through the GEO database. With *p* < 0.05 as the threshold, the GSE111412 microarray dataset contained adipose tissue samples from mice fed with a normal diet and those fed with HFD (each *n* = 10). The samples were analyzed, which obtained 1240 DEGs (Figure 2A,B). The GSE139601 dataset contained 12 cases of adipose tissue samples from mice fed with a low-fat diet (LFD) and 11 cases of adipose tissue samples from mice with HFD (LFD or HFD for 4, 8 and 12 weeks); 976 DEGs were obtained through the analysis (Figure 2C,D). The GSE15653 dataset contained liver tissue samples from 5 normal individuals and 4 obese patients, and 1652 DEGs were obtained via the analysis (Figure 2E,F). The above 3 microarray analyses were intersected, and 3376 DEGs related to acquired hyperlipidemia/obesity were thus obtained. To further combine targets related to dyslipidemia, “hyperlipidemia” was retrieved in the CTD database, and 1479 related targets were obtained using the inference score ≥ 50 as the screening condition. Finally, we intersected the GEO database’s analytical results with the CTD database’s screening results, and a total of 474 disease targets were obtained (Figure 2G).

### 3.4. GO and KEGG Enrichment Analyses of 101 Common Targets of AM and Acquired Hyperlipidemia

**Figure 3:**
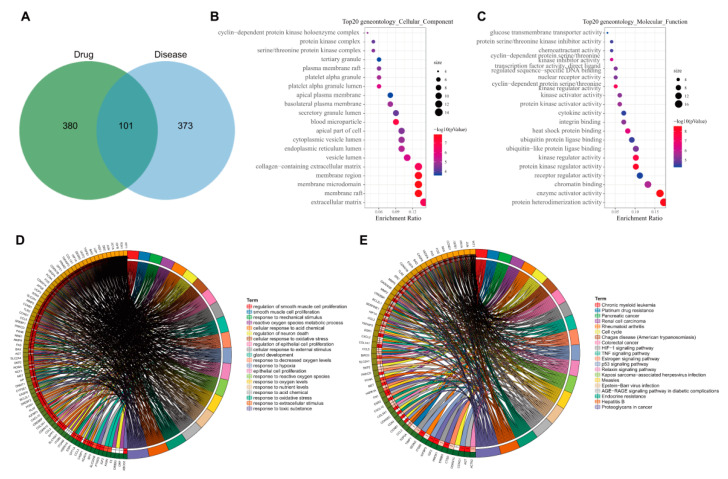
GO and KEGG analyses of 101 candidate targets. (**A**) Venn diagram of intersection of drug and disease targets. (**B**) The bubble diagram of enrichment analysis on cell components. (**C**) The bubble diagram of enrichment analysis on molecular functions. In Panel (**B**,**C**), the original point color represents the *p*-value, and the origin size represents the number of enriched targets. The X-axis represents the number of enriched targets/the number of total targets, and the Y-axis represents the pathway name. (**D**) The circle diagram of enrichment analysis on biological processes. (**E**) The circle plot of KEGG enrichment analysis, with genes on the left and pathways on the right.

To explore the possible ways of AM in the treatment of hyperlipidemia induced by HFD, we intersected 481 AM-related targets previously obtained with 474 disease targets to obtain 101 candidate targets (Figure 3A). Further gene functional enrichment analysis was performed on these 101 candidate targets. GO enrichment analysis (*p* < 0.05) showed that they mainly had a significant impact on 47 cell components such as extracellular matrix (GO: 0031012), membrane raft (GO: 0045121) and membrane microdomain (GO: 0098857) (Figure 3B), 107 molecular functions such as protein heterodimerization activity (GO: 0046982), enzyme activator activity (GO: 0008047) and chromatin binding (GO: 0003682) have significant effects (Figure 3C), and 1681 biological processes such as the response to a toxic substance (GO: 0009636), the response to extractive stimulus (GO: 0009991), and the response to oxidative stress (GO: 0006979) (Figure 3D). 

KEGG enrichment analysis (*p* < 0.05) showed that the targets mainly had significant effects on 105 signaling pathways such as proteoglycans in cancer (hsa05205), hepatitis B (hsa05161), endocrine resistance (hsa01522), AGE-RAGE signaling pathway in diabetic complications (hsa04933), Epstein–Barr virus infection (hsa05169) and p53 signaling pathway (hsa04115) (Figure 3E). Studies have shown that the advanced glycation end products (AGEs) and its receptor RAGE (receptor for AGE) path are closely related to the development of steatohepatitis induced by type II diabetes and early diabetic retinopathy [16,17]. The p53 signaling pathway plays a role in developing type II diabetes and hyperlipidemia [18,19]. Overall, endocrine resistance, AGE-RAGE in diabetes complications and p53 may be the main signaling pathways for AM to alleviate hyperlipidemia induced by HFD.

### 3.5. Construction of Network Relationship of “AM-Component-Target”

**Figure 4:**
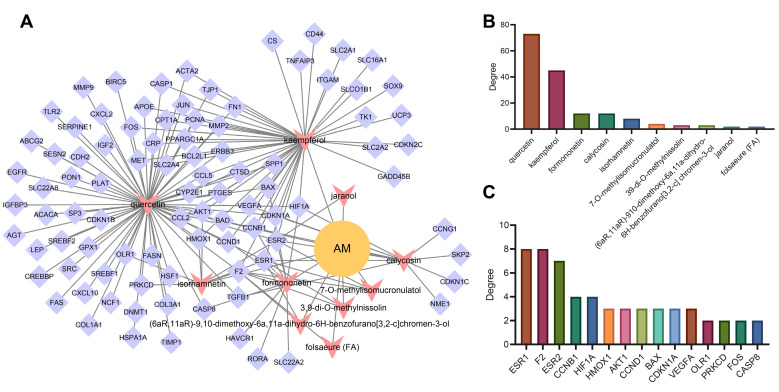
Target screening and construction of “AM-component-target” network. (**A**) “AM-component-target” network. (**B**) The ranking diagram of active components of AM according to degree value. (**C**) The ranking diagram of candidate targets according to degree value (top 15).

Based on the “AM-component-target” regulatory relationship retrieved from the TCMSP, CTD and STITCH databases, we constructed a network relationship diagram of 101 candidate genes and their corresponding active components and only retained the active components with OB ≥ 30% and DL ≥ 0.18. The “AM-component-target” network was constructed in the Cytoscape software. As shown in Figure 4A, the network involved 103 nodes containing 10 active components and 92 targets (9 targets not related to the main active components had been excluded). The degree value indicated the number of connections between a node and other nodes in the control network, which was used to quantify the core degree of the node in the interaction network. The top 15 targets were sorted according to the degree value. Meanwhile, it could be seen that quercetin, kaempferol, calycosin, formononetin and isorhamnetin may be the main active components of AM in alleviating hyperlipidemia induced by HFD (Figure 4B,C).

### 3.6. AKT1, VEGFA, CCND1 and ESR1 Were the Key Targets of AM in the Treatment of Acquired Hyperlipidemia

**Figure 5:**
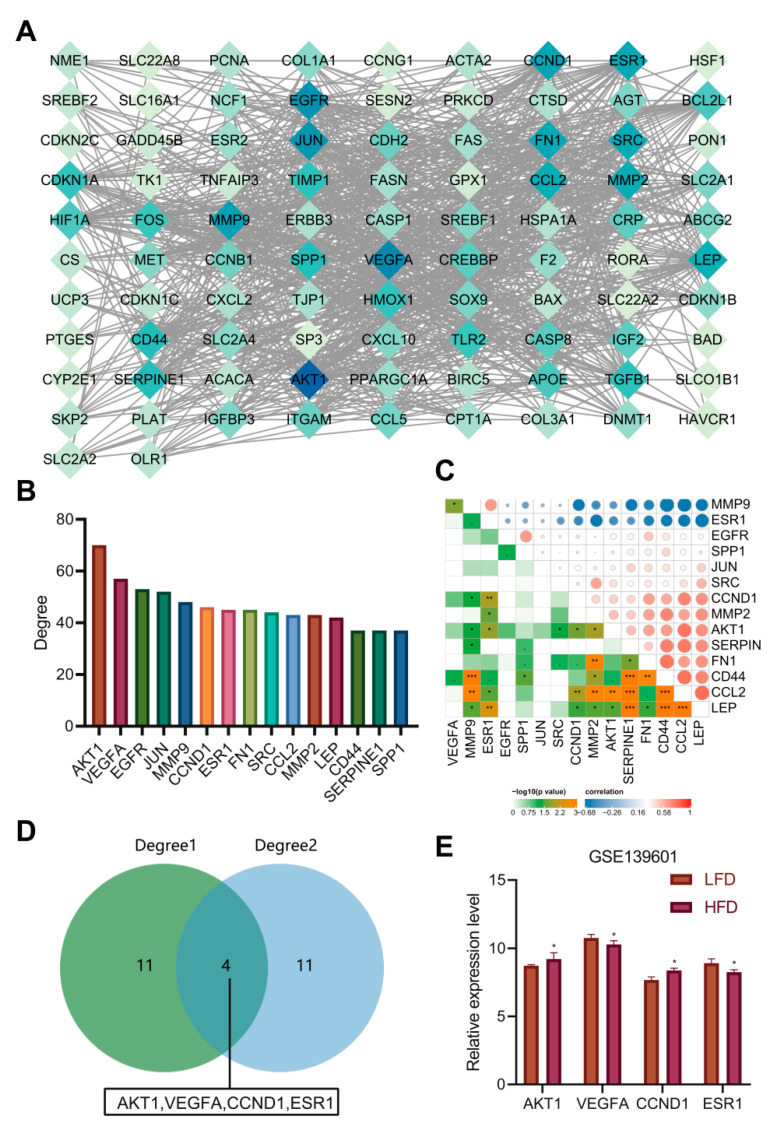
PPI analysis and determination of key targets. (**A**) The PPI diagram of 92 targets. (**B**) The ranking diagram of candidate targets according to degree value (top 15). (**C**) The correlation of the top 15 candidate targets is based on the adipose tissue sample data of 12 mice fed with LFD and 11 mice fed with HFD from the GSE139601 dataset. (**D**) Intersection of “AM-component-target” network and PPI network after degree value-based ranking. (**E**) The expression of AKT1, VEGFA, CCND1 and ESR1 in adipose tissues of mice fed with LFD and HFD based on the GSE139601 dataset. * *p* < 0.05, ** *p* < 0.01, *** *p* < 0.001.

To further explore the protein–protein interaction relationship of these 92 targets, the targets were incorporated into the STRING database (the “species” was limited to “human”) and further introduced into the Cytoscape software to construct a PPI network. As shown in Figure 5A, involving 92 nodes and 988 edges, the nodes represent proteins, the edges represent the correlation between the proteins, and the colors represent degree value. A darker color indicated a greater degree value. According to the order of degree value from large to small, the top 15 targets were selected, namely AKT1, VEGFA, epidermal growth factor receptor (EGFR), C-Jun (JUN), matrix metalloproteinase-9 (MMP9), CCND1, ESR1, fibronectin (FN1), C-Src (SRC), C-C motif chemokine ligand 2 (CCL2), matrix metalloproteinase-2 (MMP2), leptin (LEP), cluster of differentiation-44 (CD44), serine protease inhibitor clade E member 1 (SERPINE1) and secreted phosphoprotein 1 (SPP1) (Figure 5B). Based on the data of 23 samples in the GSE139601 dataset, we performed a correlation analysis on the above 15 candidate targets to further verify the PPI relationship (Figure 5C). In combination with the degree values of the 92 targets in the previous AM-component-target network, the top 15 targets were identified to be ESR1, coagulation Factor II, Thrombin (F2), estrogen receptor beta (ESR2), cathepsin D (CTSD), cyclin B1 (CCNB1), hypoxia inducible factor 1-alpha (HIF1A), proliferating cell nuclear antigen (PCNA), peroxisome proliferator-activated receptor gamma, coactivator 1 alpha (PPARGC1A), VEGFA, cyclin-dependent kinase inhibitor 1A (CDKN1A), BCL-2-associated X (BAX), CCND1, C-Fos (FOS), AKT1 and heme oxygenase 1 (HMOX1), respectively. The two gene sets were subsequently intersected to obtain four key targets (AKT1, VEGFA, CCND1 and ESR1) (Figure 5D). The expression of the four key targets in adipose tissues (GSE139601) of mice fed with LFD and HFD is displayed in Figure 5E. Compared with that in adipose tissues of mice fed with LFD, the expression of AKT1 and CCND1 in those of mice fed with HFD was significantly upregulated, while the expression of VEGFA and ESR1 was significantly downregulated.

According to previous literature, the deletion of AKT1 increased energy consumption and prevented diet-induced obesity in mice [20]. Poor vascularization can lead to fibrosis and local inflammation related to insulin resistance during adipose tissue hyperplasia. VEGFA has been important in promoting angiogenesis, decreasing inflammation, and increasing adipose tissue function [21]. Meanwhile, ESR1 activator in mouse adipose tissues can induce the expression of VEGFA [22]. ESR1 expression in adipose tissues was negatively correlated with obesity and genes related to mitochondrial metabolism and metabolic health markers [23]. In addition, CCND1 increased significantly in diabetic patients’ liver and was one of the most significant genes in obesity/diabetes liver tumors [24]. So far, we believe that AM may alleviate hyperlipidemia induced by HFD by downregulating the AKT1 and CCND1 protein levels and upregulating the levels of VEGFA and ESR1 protein.

### 3.7. AKT1, VEGFA, CCND1 and ESR1 Are Closely Related to Abnormal Lipid Metabolism

**Figure 6:**
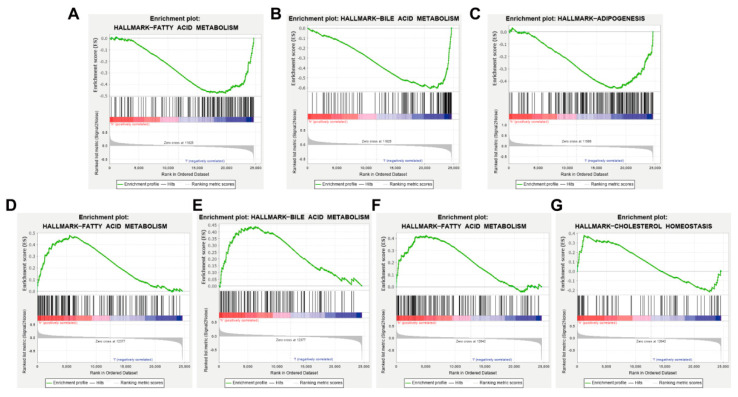
GSEA analytical results based on the high and low expression of key targets. (**A**,**B**) Enrichment of AKT1 low expression gene set in fatty acid metabolism and bile acid metabolism, respectively. (**C**) Enrichment of CCND1 low expression gene set in oxidative phosphorylation pathway. (**D**,**E**) Enrichment of VEGFA high expression gene set in fatty acid metabolism and bile acid metabolism. (**F**,**G**) Enrichment of ESR1 high expression gene set in fatty acid metabolism and cholesterol homeostasis pathway.

GSEA analysis was performed according to the high and low expression of AKT1, CCND1, VEGFA and ESR1 based on the microarray dataset GSE139601, with |NES| > 1, NOM *p*-value < 0.05 and FDR q-val < 0.25 as the threshold. The results showed that the AKT1 low expression gene set was enriched in the pathways of fatty acid metabolism and bile acid metabolism, suggesting that AKT1 might inhibit fatty acid metabolism and bile acid metabolism (Figure 6A,B). The CCND1 low expression gene set was enriched in the oxidative phosphorylation pathway, suggesting that CCND1 might inhibit oxidative phosphorylation (Figure 6C). The VEGFA high expression gene set was enriched in fatty acid metabolism and bile acid metabolism pathways, which suggested that VEGFA might promote fatty acid metabolism and bile acid metabolism (Figure 6D,E). The enrichment of the ESR1 high expression gene set was found in fatty acid metabolism and cholesterol homeostasis pathways, indicating that ESR1 might promote fatty acid metabolism and cholesterol homeostasis (Figure 6F,G). The above results also verify that AKT1, CCND1, VEGFA and ESR1 may be the key targets of AM in the treatment of acquired hyperlipidemia.

Based on the above evidence, we conclude that endocrine resistance, AGE-RAGE signaling pathway in diabetic complications and p53 signaling pathway may be the main ways AM can treat acquired hyperlipidemia. Quercetin, kaempferol, calycosin, formononetin and isorhamnetin may be the main active components of AM in the treatment of acquired hyperlipidemia. More importantly, AKT1, VEGFA, CCND1, ESR1 are the key targets of AM to alleviate hyperlipidemia induced by HFD.

### 3.8. AM Reduced the Weight Gain Caused by HFD and Alleviated Dyslipidemia

**Figure 7:**
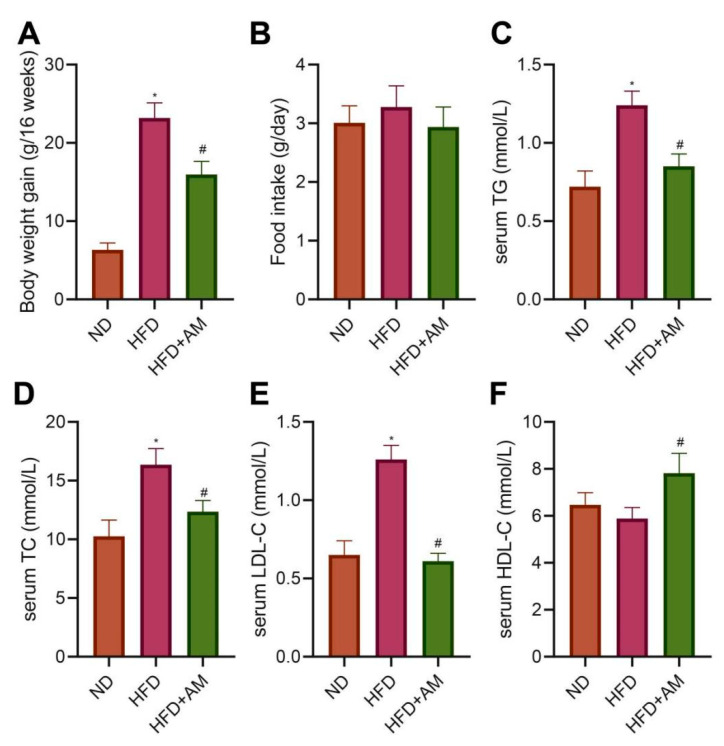
Effects of AM administration on the body weight and serum lipid profile in mice fed with HFD. (**A**) Bodyweight gain (*n* = 10). (**B**) Food intake (*n* = 10). (**C**) Serum TG (*n* = 7). (**D**) Serum TC (*n* = 7). (**E**) Serum LDL-C (*n* = 7). (**F**) Serum HDL-C (*n* = 7). Results are expressed as means ± SD. * *p* < 0.05 compared with ND group, # *p* < 0.05 compared with HFD group. ND, normal diet; HFD, high-fat diet; AM, *Astragalus membranaceus*; TG, triglyceride; TC, total cholesterol; LDL-C, low-density lipoprotein cholesterol; HDL-C, high-density lipoprotein cholesterol.

To further verify the function of AM in mice fed with HFD, we divided adult male C57BL/6J mice into the normal diet (ND group) and HFD (HFD group). After 8 weeks, hyperlipidemia mice were further treated with AM extract (HFD + AM group) and gavaged for 8 weeks. The results showed that the body weight gain in the HFD group was higher than that in the ND group. In contrast, the HFD + AM group showed a vigorous reduction in body weight gain compared to the HFD group (Figure 7A), and in food intake, no significant differences found (Figure 7B). These results suggested that, without reducing food intake, the AM treatment had diminished the problem of excessive weight gain caused by HFD in mice.

To evaluate the effects of AM on HFD-related dyslipidemia, we measured the levels of serum triglycerides (TG), total cholesterol (TC), low-density lipoprotein cholesterol (LDL-C) and high-density lipoprotein cholesterol (HDL-C) levels. The serum TG, TC, and LDL-C concentrations showed a noticeable increase in the HFD group compared with the ND group. At the same time, AM treatment significantly reduced serum TG, TC, LDL-C levels and increased serum HDL-C level (Figure 7C–F). The data demonstrated that AM attenuated HFD-related dyslipidemia in mice.

### 3.9. AM Inhibited Lipogenesis, Promoted Lipolysis and Lipid β-Oxidation in Liver Tissue and WAT of Mice Fed with HFD

**Figure 8:**
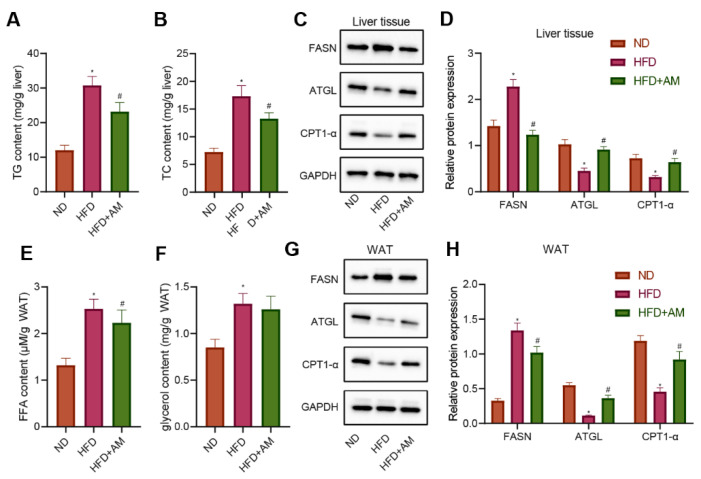
Effects of AM administration on the lipid metabolism in liver tissue and WAT of mice fed with HFD. (**A**) Liver TG content. (**B**) Liver TC content. (**C**) Representative Western blots of FASN, ATGL, CPT1-α and GAPDH proteins in liver tissue. (**D**) Density analysis of FASN, ATGL, CPT1-α based on the ratio to GAPDH in liver tissue. (**E**) FFA content in WAT. (**F**) Glycerol content in WAT. (**G**) Representative Western blots of FASN, ATGL, CPT1-α and GAPDH proteins in WAT. (**H**) Density analysis of FASN, ATGL, CPT1-α based on the ratio to GAPDH in WAT. Results are expressed as means± SD (*n* = 7). * *p* < 0.05 compared with ND group, # *p* < 0.05 compared with HFD group. FFA, free fatty acid. WAT, white adipose tissue.

To investigate AM’s potential mechanism to ameliorate the abnormal liver lipid metabolism in mice fed with HFD, we detected the liver weight, liver TC and TG levels, and the expressions of several representative lipid metabolism-related proteins in the liver tissue. Compared with the ND group, the liver TC and TG levels in the HFD group were significantly higher, while those in the HFD + AM group were significantly lower than those in the HFD group (Figure 8A,B). Meanwhile, the level of FASN for lipogenesis was diminished by AM treatment. The levels of lipolytic ATGL protein and CPT1-α protein involved in lipid β-oxidation were significantly increased after AM intervention (Figure 8C,D). These results demonstrated that AM markedly reduced the process of lipogenesis and increased the process of lipolysis and lipid β-oxidation in liver tissue.

On the other hand, we collected the WAT of mice in each group to detect the contents of free fatty acid (FFA) and glycerol. The results showed that the contents of FFA and glycerol increased significantly in the HFD group compared with the ND group, while the FFA contentin the HFD + AM group decreased significantly compared with the HFD group (Figure 8E,F). In addition, the level of FASN protein was diminished by AM treatment, and the levels of ATGL and CPT1-α protein were significantly increased after AM treatment (Figure 8G,H). The results showed that AM reduced the lipid storage and increased lipolysis and lipid β-oxidation in WAT induced by HFD.

### 3.10. AM Regulated the Expression of AKT1, VEGFA, CCND1 and ESR1 in the Liver and WAT of Mice Fed with HFD

**Figure 9:**
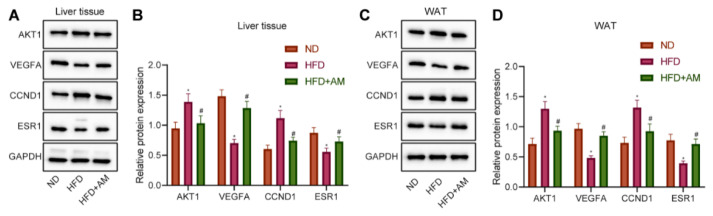
Effects of AM administration on the AKT1, VEGFA, CCND1, ESR1 protein expression in liver tissue and WAT of mice fed with HFD. (**A**) Representative Western blots of AKT1, VEGFA, CCND1, ESR1 and GAPDH proteins in liver tissue. (**B**) Density analysis of proteins based on the ratio to GAPDH in liver tissue. (**C**) Representative Western blots of AKT1, VEGFA, CCND1, ESR1 and GAPDH proteins in WAT. (**D**) Density analysis of proteins based on the ratio to GAPDH in WAT. Results are expressed as means± SD (*n* = 7). * *p* < 0.05 compared with ND group, # *p* < 0.05 compared with HFD group.

The previous bioinformatics analysis results showed that AM might alleviate hyperlipidemia by HFD through regulating the expression of four key targets (AKT1, VEGFA, CCND1 and ESR1). Therefore, we further verify whether AM can change the expression levels of the above four key targets in liver tissue and WAT of mice fed with HFD. We found that the levels of AKT1 and CCND1 protein were increased significantly, and the levels of VEGFA and ESR1 protein were decreased significantly in the HFD group compared with the ND group. Furthermore, AKT1 and CCND1 protein levels were significantly increased, and the levels of VEGFA and ESR1 protein were significantly decreased after AM administration (Figure 9). The Western blot results in WAT were completely consistent with liver tissue. So far, we believe that AM may reduce the expression of AKT1 and CCND1 and increase the expression of VEGFA and ESR1 to alleviate hyperlipidemia induced by HFD.

## 4. Discussion

AM is one of the most widely used plants with the homology of food and medicine, with multiple health-promoting effects [25]. The results presented in this study support the possibility of AM to alleviate hyperlipidemia in mice fed with HFD through regulating lipid metabolism. We reported for the first time that the effects of AM on acquired hyperlipidemia are likely achieved by regulating the lipid metabolism, it markedly reduced the process of lipogenesis, and increased the process of lipolysis and lipid β-oxidation in liver tissue and WAT; moreover, the key targets of AM might be AKT1, VEGFA, CCND1 and ESR1.

With the rapid development of chemical biology, network biology and systems biology, network pharmacology is moving forward to the cutting-edge field of natural functional compounds discovery and development. Network pharmacology combines reductionism with system theory, calculation and experiment, pays attention to the overall thinking of natural active ingredients and the transformation from single target and single drug to network target and multi-component therapy. It is worth mentioning that our research goal is also towards clinical application, so, we picked up the human genes from GEO and CTD databases. However, at the initial of the research, the clinical experiments and human trials cannot be carried out directly. It is reasonable to use this animal model to explore the potential effects of functional factors. Therefore, we did the in vivo experiment at the animal level first in order to verify the results from the bioinformatics analysis.

Based on the results obtained in the current study, endocrine resistance (hsa01522), AGE-RAGE signaling pathway in diabetic complications (hsa04933) and p53 signaling pathway (hsa04115) might be the main signaling pathways of AM in the treatment of acquired hyperlipidemia. Endocrine diseases have been highlighted as a fundamental cause of acquired hyperlipidemia [26]. It was found that active compounds in AM might mediate pancreatic β-cell metabolism, thereby improving insulin secretion [27]. To our acknowledge previous studies have shown a close relation of the AGE-RAGE pathway to the development of type II diabetes-induced steatohepatitis and early diabetic retinopathy [16,17]. Strikingly, AM could treat diabetic retinopathy through the AGE-RAGE signaling pathway in diabetic complications to oxidative stress, angiogenesis, and inflammation [28]. The functions of the p53 signaling pathway on developing type II diabetes and acquired hyperlipidemia acute pancreatitis associated with kidney injury [18,19]. Intriguingly, AM altered p53 expression to protect human endothelial cells from lipopolysaccharide-triggered apoptosis [29]. Moreover, the AM extract Astragaloside IV was revealed to regulate p53 in hepatic stellate cells [30]. The reports mentioned above could collectively support our finding indicating endocrine resistance, AGE-RAGE in diabetic complications and p53 as the main signaling pathways of AM in the treatment of acquired hyperlipidemia.

We found that AM might regulate acquired hyperlipidemia-related targets by its natural functional components. According to the number of connections with the targets, the key elements are *quercetin*, *kaempferol*, *calycosin*, *formononetin* and *isorhamnetin* in turn. Previous studies have demonstrated that *quercetin* could attenuate hyperglycemia, hyperlipidemia, and oxidative stress in a rat model of type 2 diabetes [31]. It was also found that Ginkgo biloba, which contains flavone glycosides including *kaempferol*, *quercetin* and *isorhamnetin*, could affect metabolic syndrome in the aspect of obesity, high blood pressure, dyslipidemia, as well as hyperglycemia [32]. Interestingly, active fraction treatment including *calycosin* and *formononetin* diminished fed and fasting glucose, serum triglyceride, and insulin resistance in db/db obese mice [33]. *Formononetin* has been uncovered to attenuate acquired hyperlipidemia, obesity and hepatic steatosis [34]. Additionally, *isorhamnetin* derivatives have been suggested to regulate cholesterol permeability in hypercholesterolemia [35]. Overall, AM could exert pharmaceutical effects on acquired hyperlipidemia through its active components: *quercetin*, *kaempferol*, *calycosin*, *formononetin* and *isorhamnetin*. However, the specific functions of each active components and/or the synergistic effect among them were not clear until now, which need to be explored by more relevant researches.

The effects of AM and its complications on the treatment of diabetes have been confirmed. There is clinical evidence that AM injection showed a higher impact on renal protection and improved systemic status in patients with diabetic nephropathy than the control group [36]. However, there are few studies on improving dyslipidemia or lipid metabolism by AM so far. This study found that AM treatment significantly reduced serum TG, TC, LDL-C levels and increased serum HDL-C level, demonstrating that AM attenuated HFD-related dyslipidemia in mice. It was worth mentioning that we found that AM extract could reduce the liver TC and TG levels. Meanwhile, AM extract could diminish the level of FASN for lipogenesis and increase the lipolytic ATGL protein and CPT1-α protein involved in lipid β-oxidation. Although FASN, ATGL and CPT1-α cannot completely reflect the whole triglyceride metabolism, these results indicated that AM markedly reduced the process of lipogenesis and improved the process of lipolysis and lipid β-oxidation in liver tissue of mice fed with HFD to a certain extent. Combined with the evidence, Ma, C et al. demonstrated that the flavone of AM ameliorated the hepatic steatosis and lipid disorder, which contributed to the amelioration of atherosclerosis [37]. In addition, we found that the content of FFA decreased significantly in the HFD + AM group compared with the HFD group. The level of FASN protein was diminished by AM treatment, and the levels of ATGL and CPT1-α protein were significantly increased after AM intervention. Therefore, we thought that AM decreased lipid storage increased lipolysis and lipid β-oxidation in WAT induced by an HFD in mice. Other evidence suggested that AM could reduce obesity and associated metabolic disorders by regulating adipocyte thermogenesis [38]. Thus, we believed that AM alleviated acquired hyperlipidemia by regulating lipid metabolism in liver tissue and WAT.

The results of network pharmacology and bioinformatics analysis in the current study showed that AM might alleviate acquired hyperlipidemia by downregulating the expression of AKT1 and CCND1 and upregulating the expression of VEGFA and ESR1. Previous research had shown that lack of AKT1 expression could lead to increased energy consumption while preventing diet-induced obesity [20]. AKT1 activation relies on the phosphoinositide 3-kinase (PI3K) pathway and is recognized as a critical node in the path, which plays a role in the process of endocrine resistance [39]. The biochemical parameters of glucose and lipid metabolism in diabetic rats increased to varied degrees after 12 weeks of AM extract therapy, and AKT was activated in liver tissue through phosphorylation and deacetylation [40]. Combined with the results of this study, the level of AKT1 protein was increased significantly in the HFD group compared with the ND group.

Additionally, AM administration increased the level of AKT1 protein in turn. Thus, we supposed that the regulation effect on lipid metabolism by AM might be associated with the endocrine resistance pathway through AKT1 [41]. We also found that AM administration significantly increased the level of CCND1 protein in the liver tissue and WAT of HFD-fed mice. CCND1 is a key cell-cycle regulatory protein, and overexpression of CCND1 was observed in aged non-alcoholic fatty liver disease mice [42]. In addition, CCND1 was significantly increased in the liver of diabetic patients [24]. Active fraction extracted from AM root was found to increase the expression of VEGFA, which could regulate angiogenesis in zebrafish embryos and endothelial cells proliferation [43].

Moreover, compared with the ND group, the level of VEGFA protein was decreased significantly in the HFD group, and the level of VEGFA protein was significantly reduced after AM administration. VEGFA is considered a potential genetic candidate involved in type 2 diabetes and diabetic retinopathy progression [44]. Studies by Yao, J et al. confirmed that the increased plasma level of VEGFA in blotchy marrow resulted in reduced lipid profile after HFD [45]. ESR1 shared a negative correlation with obesity in adipose tissues and a positive association with mitochondrial metabolism-related genes and metabolic health markers [23]. Of note, there was protein interaction between ESR1 and p53 [46]. ESR1 was reported to be the key target of AM in the treatment of type 2 diabetes mellitus [47]. The results of this study were consistent with the above studies. We found that AM might alleviate lipid metabolism in HFD-fed mice by reducing the level of ESR1 protein. Supportably, it could be seen that AKT1 and CCND1, VEGFA and ESR1 were involved in hyperlipidemia-related diseases and several studies had uncovered the regulation of AM on these genes in different diseases. Nevertheless, the specific mechanism of AM in acquired hyperlipidemia induced by HFD remains to be explored. It is necessary to further design rescue experiments on mice, human liver cells and adipocytes to clarify the specific mechanism of AM regulating key targets (AKT1, VEGFA, CCND1 and ESR1), which is also our future research direction. Since hyperlipidemia is one of the important risk factors of insulin resistance and related metabolic diseases, if AM can be proved to reduce serum lipid and improve lipid metabolism in human beings, it will act as a candidate for treating multiple nutritional noncommunicable diseases.

## 5. Conclusions

In conclusion, AM alleviated acquired hyperlipidemia in mice fed with HFD through regulating lipid metabolism, and AKT1, VEGFA, CCND1 and ESR1 may be the key targets. The present study results contribute to a better understanding of the effects of AM on acquired hyperlipidemia and explore the key targets of natural functional components in the treatment of acquired hyperlipidemia.

## Figures and Tables

**Table 1:** Twenty active components of AM.

Mol ID	Molecule Name	OB (%)	DL
MOL000398	*Isoflavanone*	109.99	0.30
MOL000378	*7-O-methylisomucronulatol*	74.69	0.30
MOL000392	*Formononetin*	69.67	0.21
MOL000433	*Folsaeure (FA)*	68.96	0.71
MOL000438	*(3R)-3-(2-hydroxy-3,4-dimethoxyphenyl) chroman-7-ol*	67.67	0.26
MOL000380	*(6aR,11aR)-9,10-dimethoxy-6a,11a-dihydro-6H-benzofurano[3,2-c] chromen-3-ol*	64.26	0.42
MOL000211	*Mairin*	55.38	0.78
MOL000371	*3,9-di-O-methylnissolin*	53.74	0.48
MOL000239	*Jaranol*	50.83	0.29
MOL000354	*Isorhamnetin*	49.60	0.31
MOL000439	*Isomucronulatol-7,2*′*-di-O-glucosiole*	49.28	0.62
MOL000417	*Calycosin*	47.75	0.24
MOL000098	*Quercetin*	46.43	0.28
MOL000422	*Kaempferol*	41.88	0.24
MOL000374	*5*′*-hydroxyiso-muronulatol-2*′*,5*′*-di-O-glucoside*	41.72	0.69
MOL000442	*1,7-Dihydroxy-3,9-dimethoxy pterocarpene*	39.05	0.48
MOL000296	*Hederagenin*	36.91	0.75
MOL000379	*9,10-dimethoxypterocarpan-3-O-β-D-glucoside*	36.74	0.92
MOL000033	*(3S,8S,9S,10R,13R,14S,17R)-10,13-dimethyl-17-[(2R,5S)-5-propan-2-yloctan-2-yl]-2,3,4,7,8,9,11,12,14,15,16,17-dodecahydro-1H-cyclopenta[a]phenanthren-3-ol*	36.23	0.78
MOL000387	*Bifendate*	31.10	0.67

Note: OB, oral bioavailability; DL, drug-likeness; The italics indicate the scientific names of plant compounds.

## Data Availability

The authors declare that the data supporting the findings of this study are available within the article. All components and targets of AM from TCMSP database are available in supplementary file S1. The gene expression matrix from GEO database, which was used to screen DEGs in this study, was stored as supplementary file S2. And the input and output files in GO and KEGG enrichment analysis are openly available in supplementary file S3. The input files of four genes in GSEA software can be found in supplementary file S4.

## Supplementary Materials

The following supporting information can be downloaded at: https://www.mdpi.com/article/10.3390/nu14050954/s1, Supplementary file S1. All components and targets of AM from TCMSP database. Supplementary file S2. The gene expression matrix from GEO database. Supplementary file S3. GO and KEGG enrichment analysis. Supplementary file S4. GSEA of four key genes.
