# Supplementary figures and images for: Mechanism of Astragalus membranaceus Alleviating Acquired Hyperlipidemia Induced by High-Fat Diet through Regulating Lipid Metabolism
Source: Nutrients. 2022 Feb 23;14(5):954. doi: 10.3390/nu14050954 (PMC8912611; doi:10.3390/nu14050954)

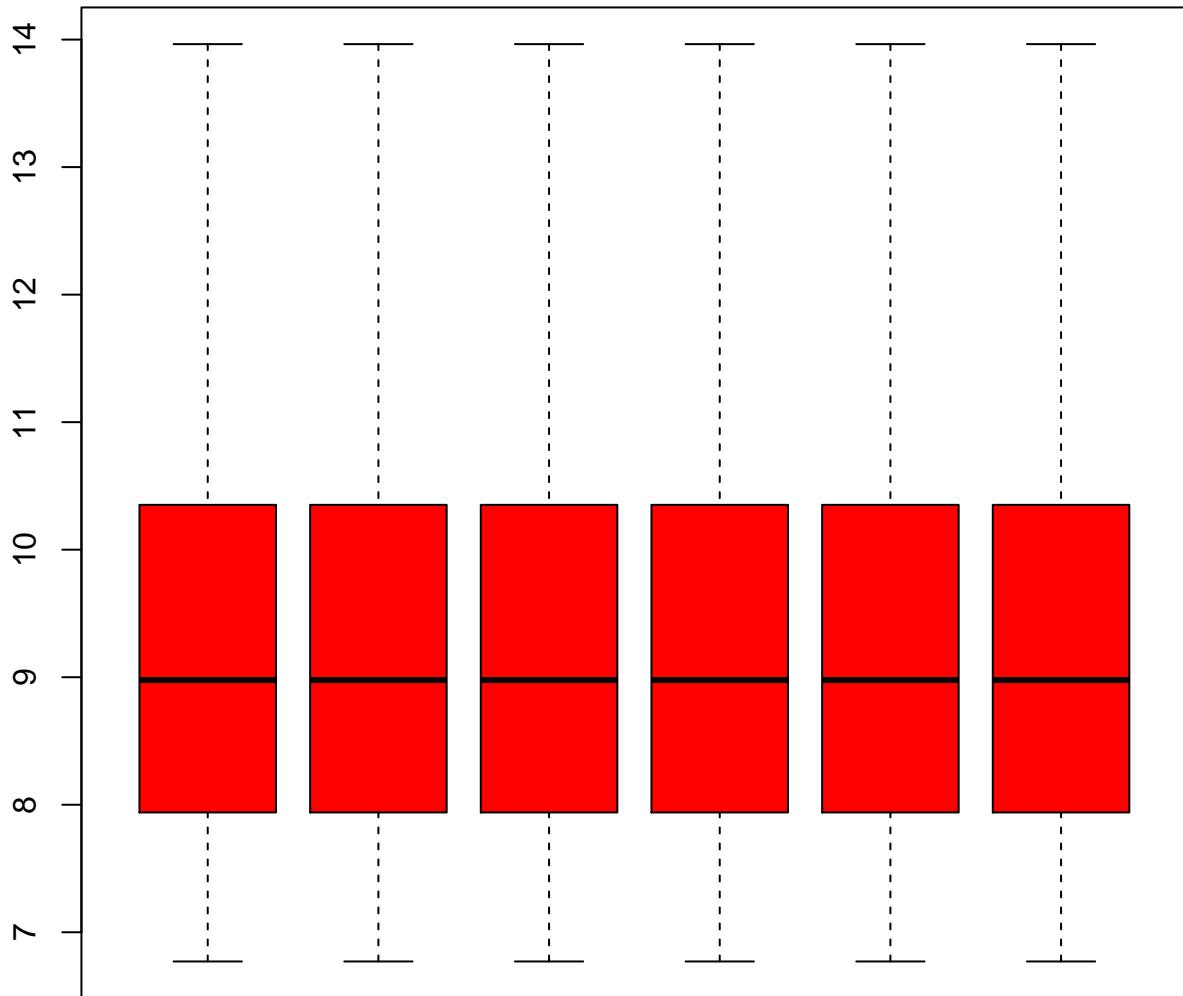

Supplement: Supplementary file 1 [file nutrients-14-00954-s001.zip › nutrients-1560760 - supplementary/supplementary file 2. The gene expression matrix from GEO database/GSE111412/DEGs/normalBox.pdf]

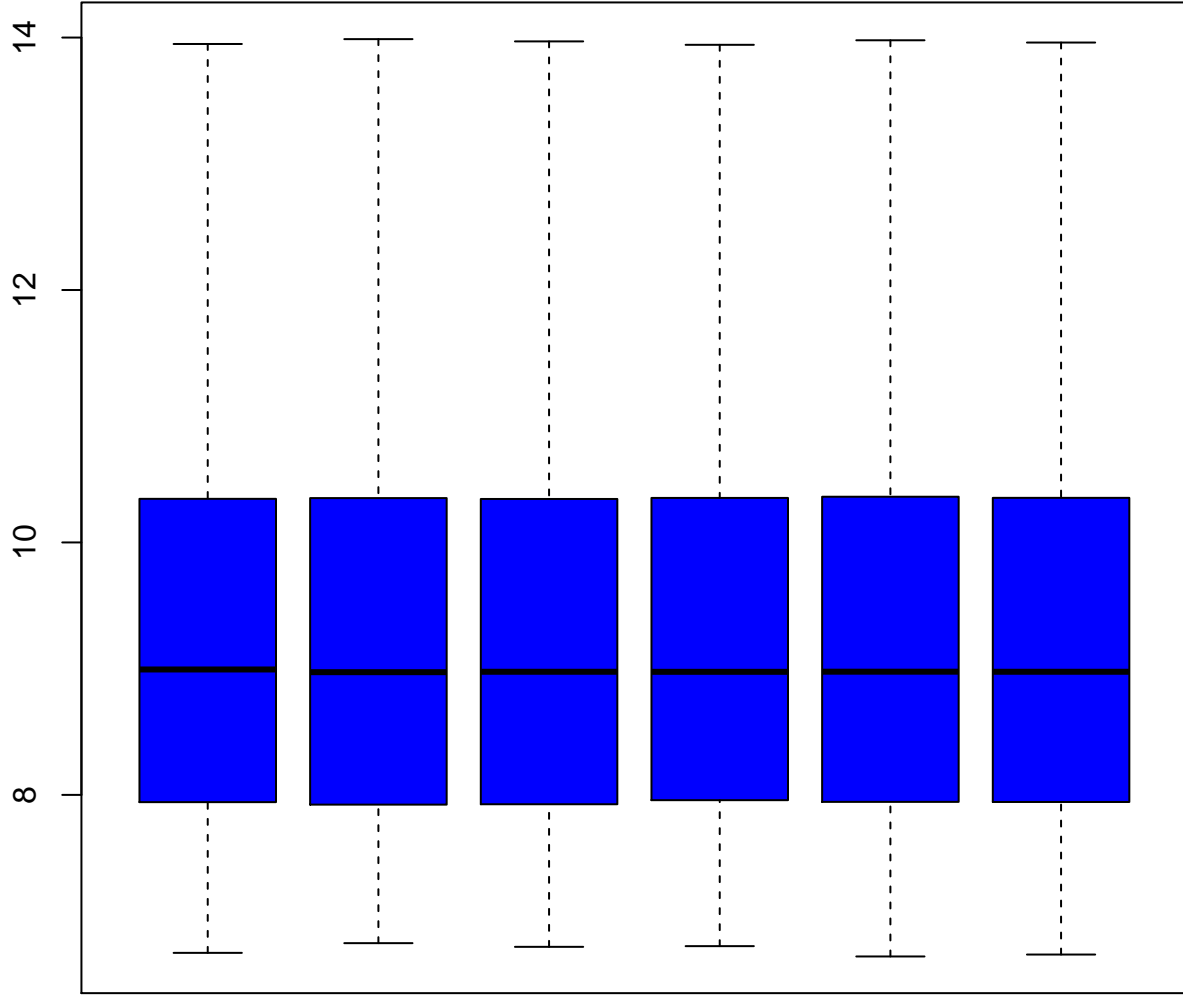

Supplement: Supplementary file 1 [file nutrients-14-00954-s001.zip › nutrients-1560760 - supplementary/supplementary file 2. The gene expression matrix from GEO database/GSE111412/DEGs/rawBox.pdf]

# Volcano

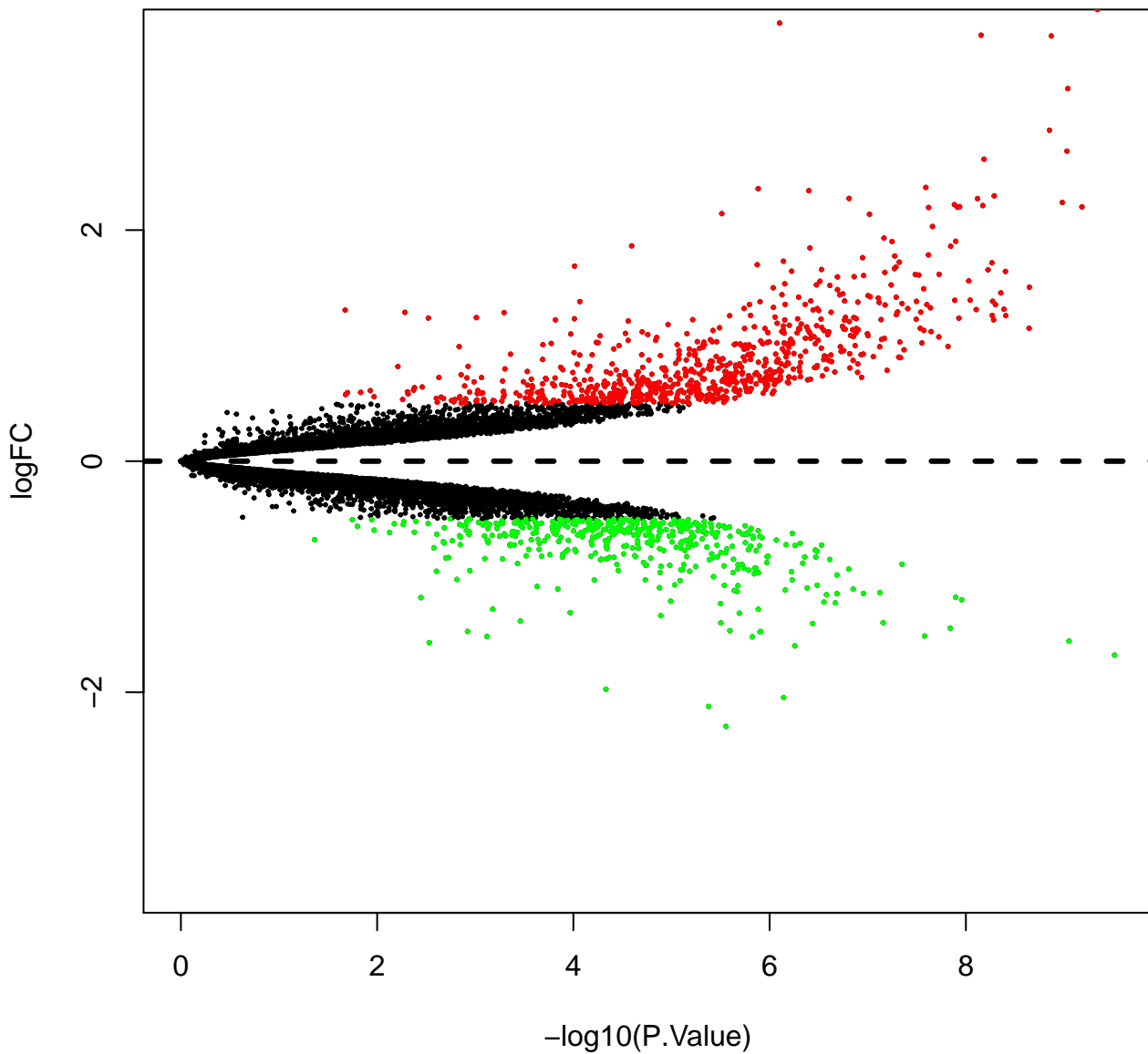

Supplement: Supplementary file 1 [file nutrients-14-00954-s001.zip › nutrients-1560760 - supplementary/supplementary file 2. The gene expression matrix from GEO database/GSE111412/DEGs/vol.pdf]

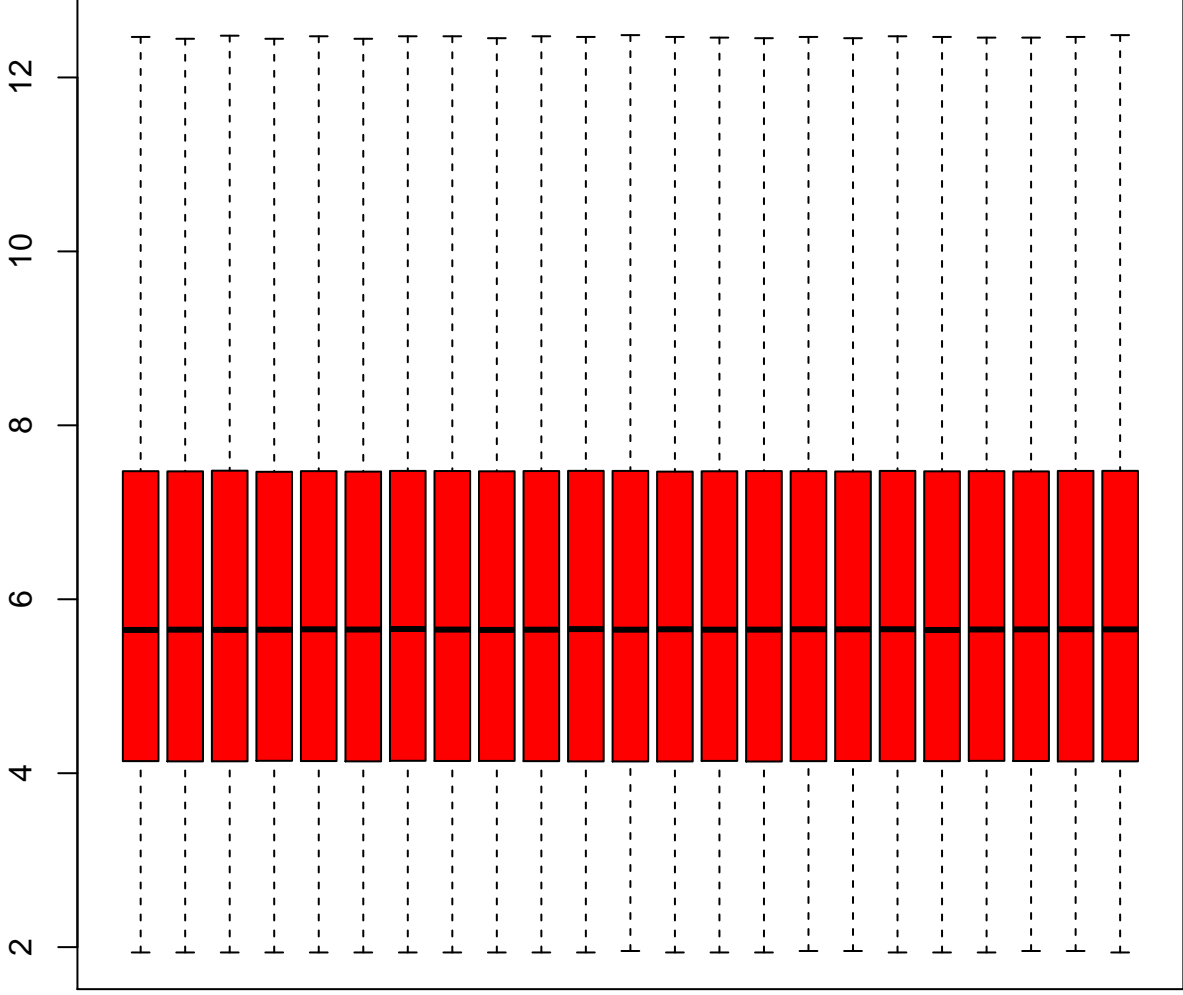

Supplement: Supplementary file 1 [file nutrients-14-00954-s001.zip › nutrients-1560760 - supplementary/supplementary file 2. The gene expression matrix from GEO database/GSE139601/DEGs/normalBox.pdf]

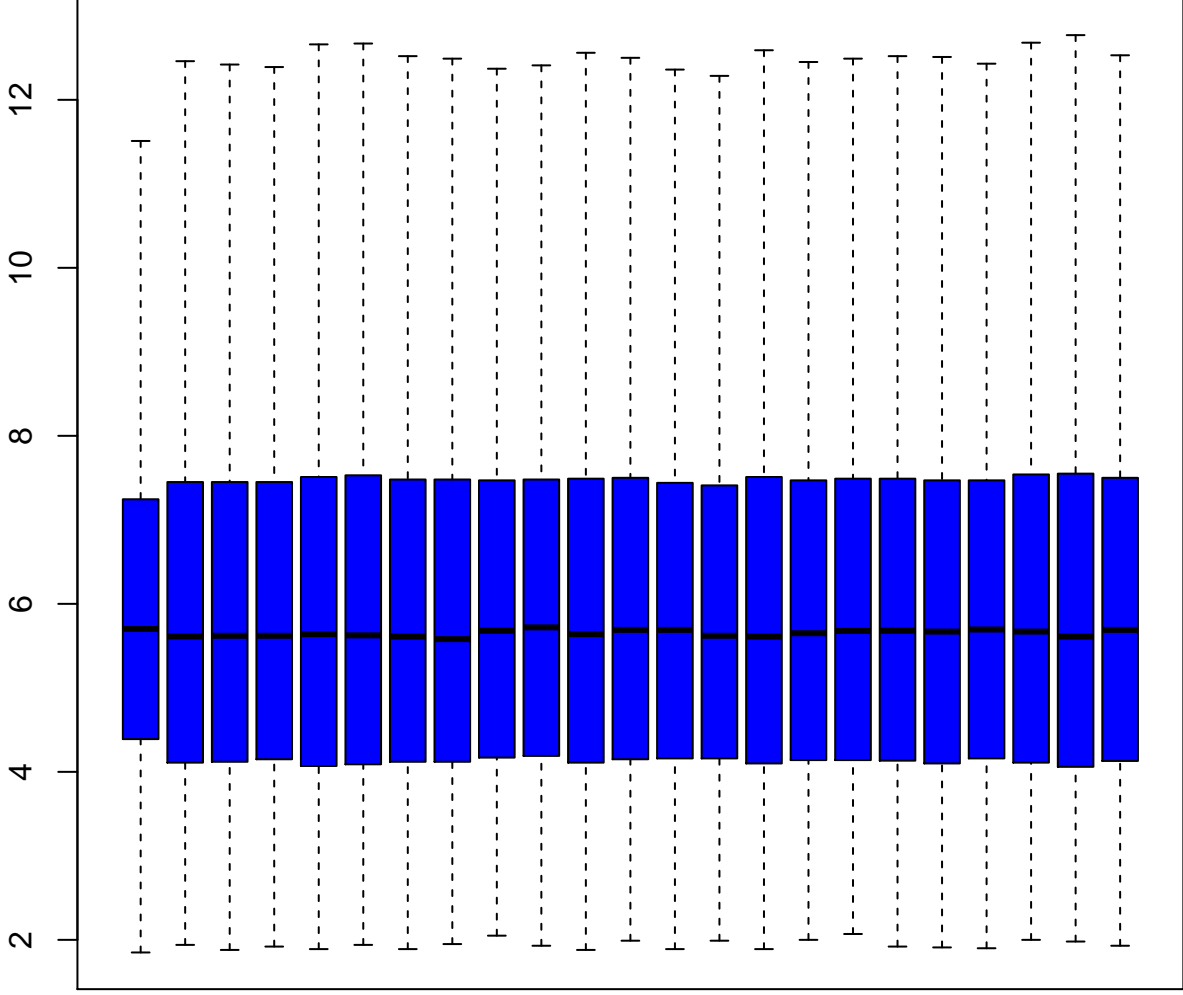

Supplement: Supplementary file 1 [file nutrients-14-00954-s001.zip › nutrients-1560760 - supplementary/supplementary file 2. The gene expression matrix from GEO database/GSE139601/DEGs/rawBox.pdf]

# Volcano

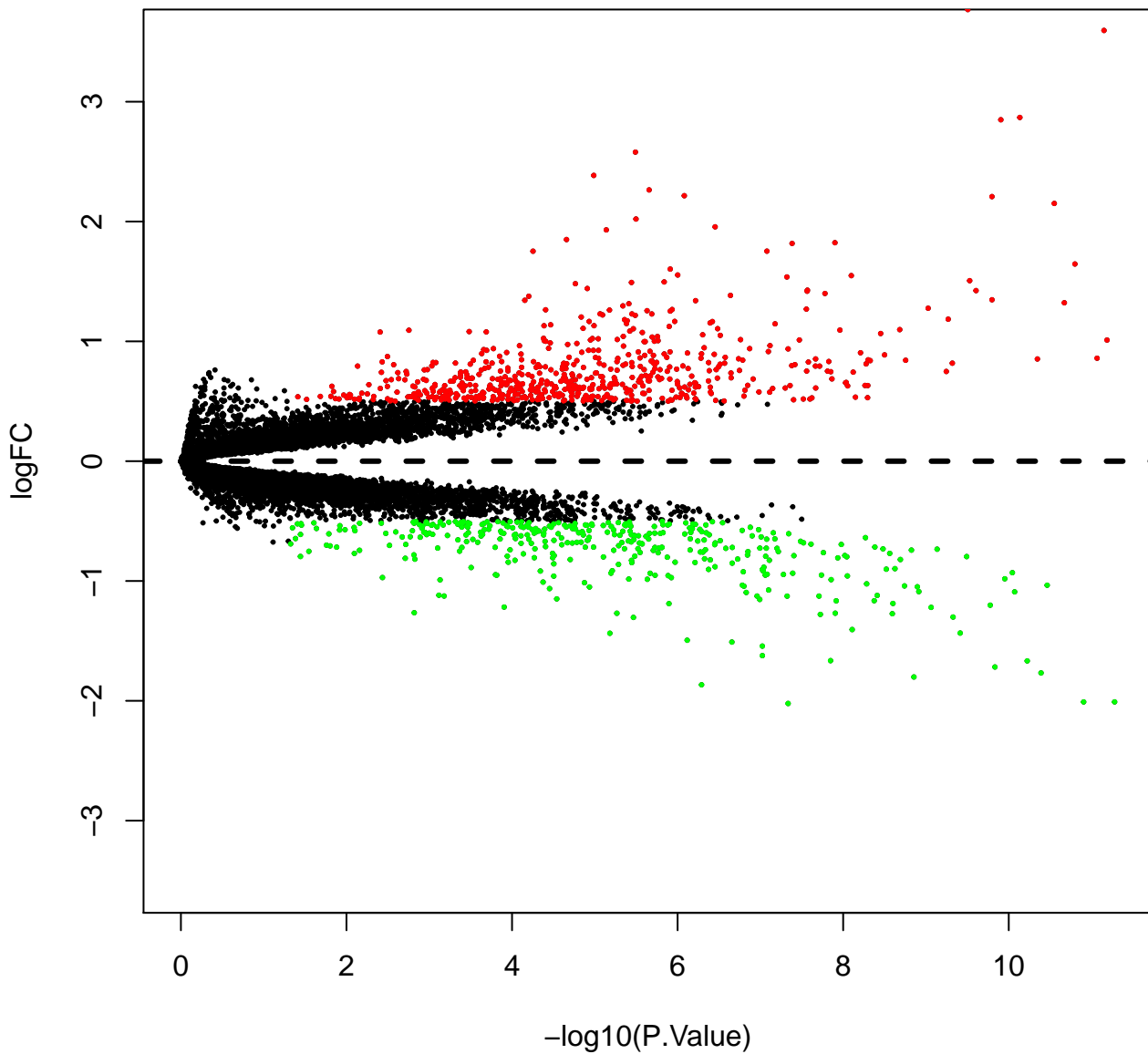

Supplement: Supplementary file 1 [file nutrients-14-00954-s001.zip › nutrients-1560760 - supplementary/supplementary file 2. The gene expression matrix from GEO database/GSE139601/DEGs/vol.pdf]

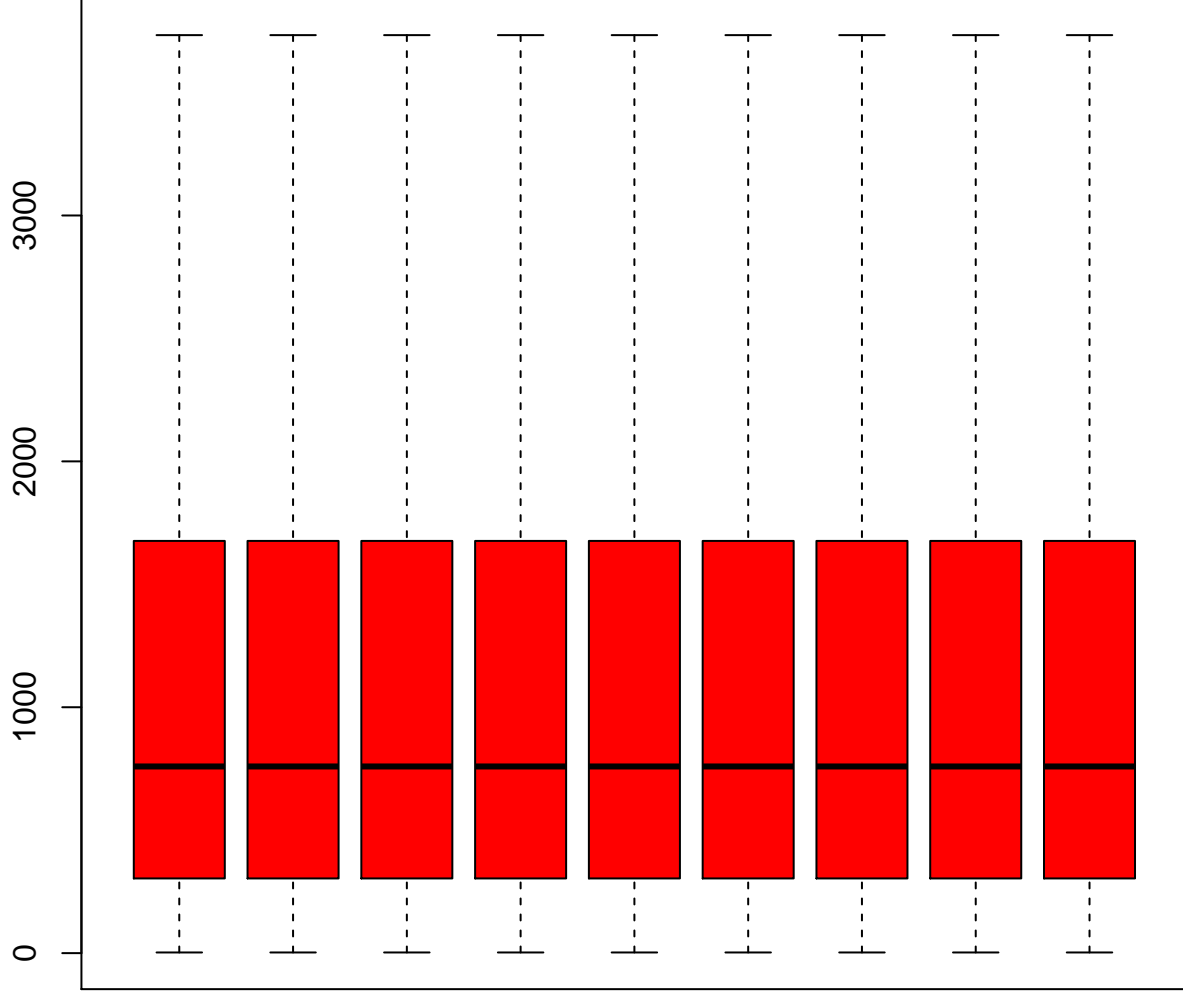

Supplement: Supplementary file 1 [file nutrients-14-00954-s001.zip › nutrients-1560760 - supplementary/supplementary file 2. The gene expression matrix from GEO database/GSE15653/DEGs/normalBox.pdf]

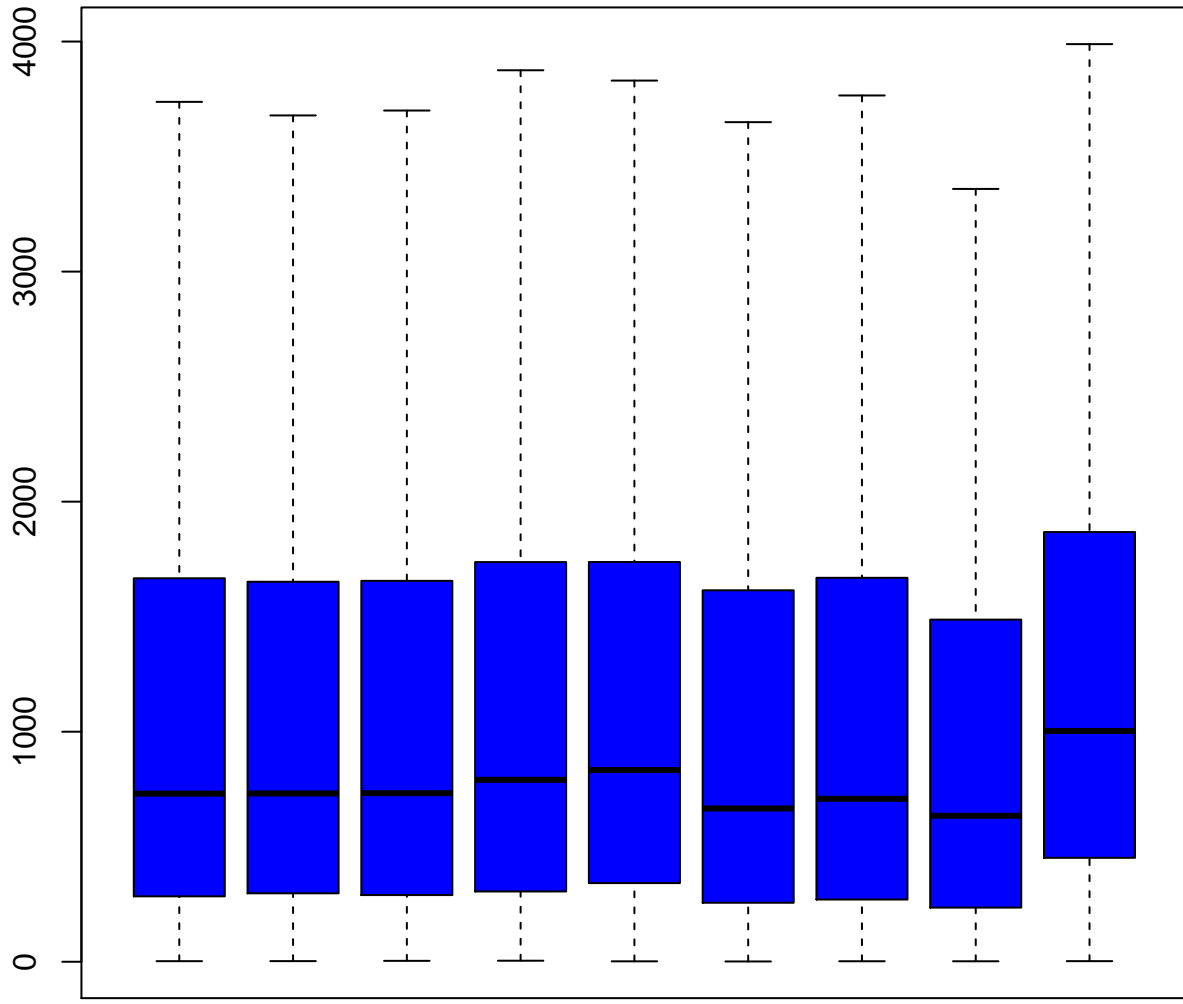

Supplement: Supplementary file 1 [file nutrients-14-00954-s001.zip › nutrients-1560760 - supplementary/supplementary file 2. The gene expression matrix from GEO database/GSE15653/DEGs/rawBox.pdf]

# Volcano

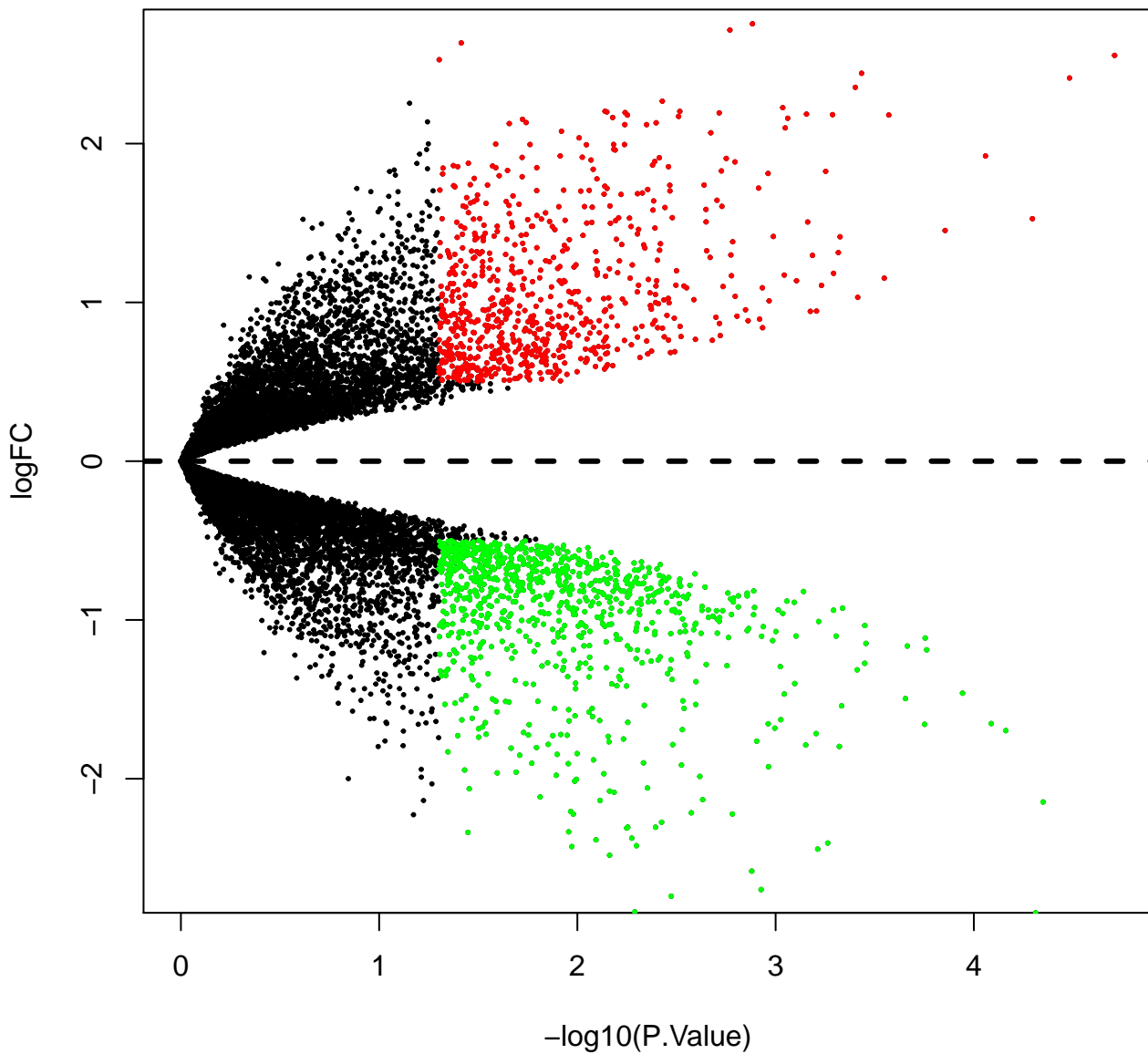

Supplement: Supplementary file 1 [file nutrients-14-00954-s001.zip › nutrients-1560760 - supplementary/supplementary file 2. The gene expression matrix from GEO database/GSE15653/DEGs/vol.pdf]
